# Review of the Use of Medicinal Cannabis Products in Palliative Care

**DOI:** 10.3390/cancers16071412

**Published:** 2024-04-04

**Authors:** James Troyer, Kimberson Tanco

**Affiliations:** Department of Palliative, Rehabilitation and Integrative Medicine, The University of Texas MD Anderson Cancer Center, Houston, TX 77030, USA; jtroyer@mdanderson.org

**Keywords:** cannabis, cannabinoids, palliative care, cancer-related symptoms, cannabis-based products, cannabis-based medicines, medical cannabis

## Abstract

**Simple Summary:**

Symptom management is a key goal of palliative cancer care. There is growing interest in the use of cannabis-based medicines or products for the management of physical and psychosocial symptoms associated with cancer or cancer treatment, including pain, nausea and vomiting, insomnia, and mood disorders. However, research on medical uses of cannabis has been limited because of legal restrictions and stigma. The goal of this review is to explore the potential role of cannabis and cannabinoids in the management of cancer-related symptoms. Limited evidence suggests that cannabis may improve cancer-related pain, chemotherapy-induced nausea and vomiting, appetite and chemosensory changes, insomnia, and mood disorders; however, cannabis is associated with several neuropsychiatric and systemic adverse effects and drug interactions.

**Abstract:**

In this review, we discuss the potential role of cannabis and cannabinoids in the management of cancer-related symptoms. There is limited evidence demonstrating the effectiveness of cannabis-based products in treating cancer-related pain and gastrointestinal symptoms such as nausea, vomiting, and loss of appetite. Regarding the role of cannabis-based products in the treatment of insomnia and mood disorders, most studies looked at these symptoms as secondary outcomes with mixed results. Cannabis-based products have adverse effects, ranging from neuropsychiatric to systemic effects to potential drug interactions.

## 1. Introduction

**Figure 1:**
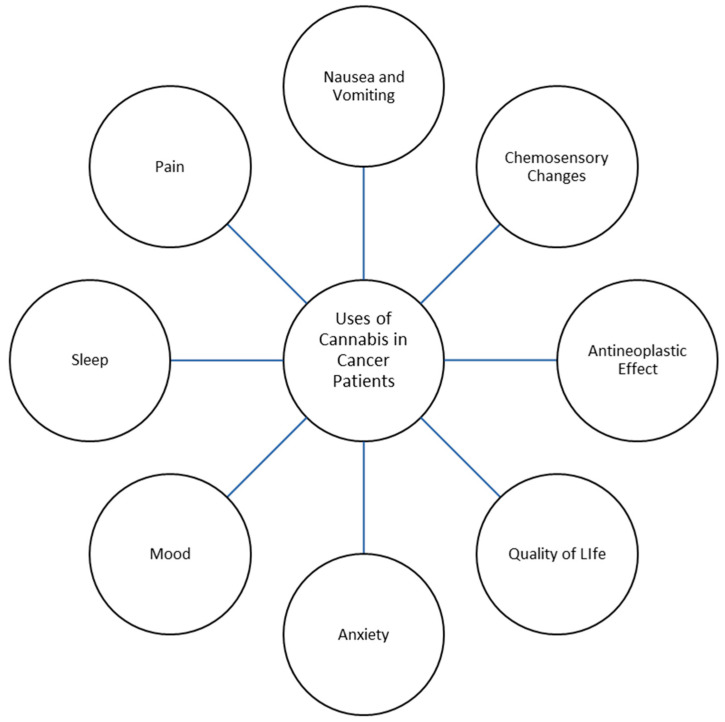
Uses of Cannabis in Cancer Patients.

Palliative care is a growing field of medicine in modern health care that strives to assuage suffering and improve the quality of life of individuals grappling with serious, life-limiting illnesses. Palliative care prioritizes holistic patient well-being through various therapeutic interventions that address symptom management rather than the treatment of an underlying condition. Patients receiving palliative care experience an array of distressing symptoms related to their illness, including pain, nausea and vomiting, loss of appetite, insomnia, and psychological distress, all of which can reduce quality of life [1,2]. Research has recently focused on the use of cannabis and the cannabinoids tetrahydrocannabinol (THC) and cannabidiol (CBD) in palliative care. *Cannabis sativa*, commonly known as cannabis, has been used medically since approximately 400 AD in areas such as China, India, and the Middle East [3,4]. The medicinal parts of *Cannabis sativa* are the plant’s flowering tops, which produce resin glands that contain cannabinoids and terpenes; other parts of the plant are used in the form of hemp fiber, hemp seeds, and other products. The plant’s flowering top is prepared and administered to the human body by various routes (Table 1). Presumed benefits from cannabis range from pain relief to antineoplastic effects, with the various literature describing cannabis being used for the treatment of chronic pain conditions, sleep and mood disorders, post-traumatic stress disorder, gastrointestinal symptoms, and neurological conditions such as Parkinson’s disease and multiple sclerosis, among others [5,6,7,8] (Figure 1). Cannabis has been previously studied as a potential addition to the repertoire of treatments for not only physical symptoms, but also psychological symptoms experienced by patients with cancer [9,10].

The available research on medical cannabis use focuses on symptom management, but cannabis use and research has been limited because of legal restrictions and cannabis’ previous stigma as a drug of abuse. Cannabis is classified as a Schedule I drug, which the US Drug Enforcement Administration defines as a drug with no currently accepted medical use and with a high potential for abuse [11]. In the United States, there are three Food and Drug Administration approved cannabinoid products: Nabilone (Schedule II), Dronabinol (Schedule III), and CBD (Schedule V) [11]. The liquid cannabis extract Nabixmols (used for spasticity or neuropathic pain associated with multiple sclerosis and cancer) is unavailable in the United States. Even though cannabis use is illegal on the federal level, medical and recreational cannabis use is legal in several states [12]. There is also a trend towards decriminalization of cannabis possession at the state and federal levels.

Patients with cancer may receive life-prolonging or life-sustaining therapies that can cause various symptoms and increased symptom burden and impair their quality of life, but they can also receive palliative care. Cannabis-based and cannabinoid medications have been studied to see if these medications can relieve common cancer-related symptoms and cancer-related therapy’s adverse effects, and thus improve quality of life [13,14]. In this review, we describe the role of cannabis and cannabinoids in the management of cancer-related symptoms.

## 2. Cancer-Related Pain

Cancer-related pain is one of the most distressing conditions patients with cancer encounter [15]. In a recent systematic literature review and meta-analysis, the overall prevalence of pain in patients with cancer was almost half, and almost one-third experience moderate to severe pain [16]. In 2023, the American Society of Clinical Oncology (ASCO) recommended that “opioids should be offered to patients with moderate-to-severe pain related to cancer or active cancer treatment unless contraindicated” [17]. Despite these recommendations, patients may fear the risks of using opioids, and clinicians may also be afraid of the consequences of prescribing opioids. This fear has led to patients finding opioid-sparing alternatives, such as cannabis-based products, to treat their cancer-related pain [18,19].

Cannabis-based medicines reduce pain through interaction with the endocannabinoid system. In the event of stress or pain, endogenous cannabinoids or introduction of exogenous cannabinoid ligands generate temporary antinociceptive effects through inhibition of presynaptic CB_1_ receptors [20]. In studies on rodents, endocannabinoids were shown to be involved in the initiation of pain, the resolution of tonic pain, and stress-induced and fear-conditioned analgesia [20].

In a study by Noyes et al. on advanced cancer patients, THC at higher doses (15–20 mg) provided better pain relief than lower doses (5–10 mg) and placebo [21]. However, the study also showed that adverse effects, including sedation and euphoria, were more prominent at higher doses of THC (15–20 mg) compared to lower doses of THC (5–10 mg) [21]. A multicenter, double-blind, randomized, placebo-controlled, parallel-group study looking at THC:CBD extract, THC extract, and a placebo found that THC:CBD extract was efficacious for pain relief in patients with advanced cancer pain that was not fully relieved with strong opioids [22]. They also found patients had worse memory- and appetite-related adverse effects with THC:CBD extract and THC extract, and worse nausea with THC:CBD extract [8]. However, similar studies did not reproduce the same outcomes of pain relief observed by Johnson et al. [23,24,25,26,27].

Due to scant evidence endorsing the utilization of cannabis-based medicines as a primary or supplementary approach for pain management, various organizations discourage the use of such medicines or products in these capacities [8,28]. Clinicians are advised to exercise caution and thoroughly evaluate the potential risks of harm and adverse events before recommending cannabis-based treatments to patients [27,29,30]. However, even with these recommendations, pain continues to be one of the most common reasons that cancer patients take cannabis-based medicines for [31,32].

## 3. Chemotherapy-Induced Nausea and Vomiting

Nausea and vomiting, specifically chemotherapy-induced nausea and vomiting (CINV), can be significant symptoms in patients with cancer [33]. Nausea and vomiting in general can be so burdensome that it leads to patients foregoing or missing their oncologic treatments [33,34]. Even with the evolving management of chemotherapy-induced nausea and vomiting, standard therapies at times are not effective in relieving nausea and vomiting. In turn, patients search for other options to adequately relieve their symptoms.

Multiple randomized controlled trials have looked at cannabis’ role in treating chemotherapy-induced nausea and vomiting. In one such trial published in 1975, Sallan et al. compared oral THC with a placebo. They found that more patients receiving THC had a complete or partially positive response than patients receiving the placebo [35]. Adverse effects noted in the study were mood changes in 81% of patients, somnolence in 66% of patients, and other toxic effects in 9% of patients (visual distortions in one patient and visual hallucinations in one patient) [35]. In 1979, Chang et al. compared oral and inhaled THC with a placebo and found that THC significantly reduced the number of vomiting and retching episodes, degree of nausea, duration of nausea, and volume of emesis in patients receiving methotrexate [36]. A few years later, Chang et al. were not able to reproduce the same results in patients receiving doxorubicin and cyclophosphamide: only three of eight patients experiencing nausea and vomiting had a fair response to oral and inhaled THC, and the other five patients had no response to oral and inhaled THC [37]. In a double-blind, placebo-controlled crossover trial comparing THC with a placebo in eleven patients, although THC showed antiemetic effects, certain patients in the THC arm dropped out of the study because of severe adverse effects and were noted to prefer nausea and vomiting to the adverse effects from THC, including dizziness, somnolence, reduced concentration, and depersonalization [38].

Several studies have compared cannabis-based medicines with other treatments for nausea and vomiting. In a systematic review conducted by Ben Amar et al., it was determined that Nabilone demonstrated notable superiority in comparison to prochlorperazine, domperidone, and alizapride in the management of nausea and vomiting related to cancer chemotherapy [39]. Dronabinol was shown to be equivalent to or significantly better than chlorpromazine and equivalent to metoclopramide, thiethylperazine, and haloperidol for treating nausea and vomiting [40]. Levonantradol, which is a synthetic cannabinoid analog of dronabinol, was found to better relieve nausea and vomiting than chlorpromazine in studies published between 1975 and 1997 [40]. Grimison et al. demonstrated that the use of THC:CBD extract led to a definite enhancement, characterized by an absence of vomiting and the absence of rescue medication usage. This was accompanied by a significant decrease in both the average and maximum occurrences of daily vomiting episodes, as well as self-reported average and maximum nausea scores when compared to a placebo [41]. In a study looking at 15mg oral THC and THC placebo smoked marijuana vs. oral THC placebo and active cigarettes, it showed 45% of patients having no preference between smoked marijuana and oral THC [42].

Several of the studies on nausea and vomiting are older and had small sample sizes. Further, to our knowledge, there are no published studies comparing cannabis-based medicines with other standard antiemetics such as 5-HT3 receptor antagonists, newer therapies like neurokinin-1 receptor antagonists, and combination anti-emetic treatment regimens. Studies have generally shown that cannabis-based medicines are more effective than placebos and similar to prochlorperazine. Adverse effects commonly reported in these studies include sedation, loss of emotional and/or physical control, nervousness, rebound nausea, somnolence, and depersonalization [24,26,27,28,29,30,31,32,33,34]. In several studies, patients withdrew, or the study was discontinued because of adverse effects [43,44,45,46,47,48].

In the consensus study report of the National Academies of Sciences, Engineering and Medicine published in 2017, the authors noted conclusive evidence supporting the use of nabilone and dronabinol for chemotherapy-induced nausea and vomiting [7]. The Multinational Association of Supportive Care in Cancer (MASCC) recommended use of cannabinoids only for refractory CINV [27]. On the other hand, the MASCC guidelines did not recommend the use of cannabinoids as the first line treatment for CINV, for the treatment of nausea and vomiting that is not secondary to chemotherapy, and the preventative treatment of radiation-induced nausea and vomiting [27]. Recently published ASCO guidelines on the use of cannabis and cannabinoids on cancer patients suggested that oral THC:CBD extracts may be considered for adult cancer patients who are receiving moderate to severely emetogenic chemotherapy regimens and have refractory CINV in spite of being on appropriate antiemetic medications [8]. They cited a phase II/III placebo-controlled, randomized, multi-center trial where patients taking THC:CBD capsules demonstrated a complete response of 24% vs. 8% for those taking the placebo [49].

## 4. Appetite and Chemosensory Changes

Eating and nutrition is a huge part of an individual’s life, and appetite impairment can decrease quality of life [50,51]. In two studies looking at patients with advanced cancer, patients receiving THC had an increase in appetite [52,53]. However, patients receiving THC had less appetite stimulation than those receiving megestrol monotherapy (75% vs. 49%), and weight gain was higher in the megestrol monotherapy group than the THC group (11% vs. 3%) [52,53]. A combination of megestrol and THC produced no significant improvement in either appetite or weight over megestrol monotherapy. In a study by the Cannabis-In-Cachexia-Study-Group, Dronabinol (2.5 mg) with or without CBD (1 mg) was compared with a placebo in patients with cancer, and no significant improvement in appetite, body weight, nausea, or quality of life was found [54]. In a double-blind randomized controlled trial in patients with cancer, Nabilone was compared with a placebo over eight weeks of treatments; the Nabilone group had no significant improvement in appetite, but had an increase in carbohydrate intake [55].

Many other studies looking at cannabis’ role in appetite and weight concerns were conducted in patients with HIV and AIDS. As compared to cancer patients, patients with HIV and AIDS receiving cannabinoids showed an improvement in appetite and either weight stabilization or weight gain compared with those receiving a placebo [56,57,58].

Chemosensory changes affect an individual’s appetite and occur during the course of the disease or cancer-directed therapy. In a double-blind randomized study of adult patients with advanced cancer in two centers, patients were given 2.5 mg of THC or placebo twice a day, and patients receiving THC were found to have improvements in chemosensory perception, taste of food, and pre-meal appetite, and an increased protein intake [59]. However, less than 50% of patients completed the trial due to being lost to follow-up or because they had experienced adverse events [59]. While the results of this study are promising, the study is not sufficient to support the use of cannabinoids for chemosensory disturbance due to low study population, and more research is needed to reproduce the results regarding the significant improvements in chemosensory perception [27,59].

## 5. Insomnia and Mood Disorders

Cancer patients, including those receiving cancer therapy, can experience impaired sleep and mood-related disorders [60,61,62]. In the literature, most studies on cannabis-based medicines had sleep and mood-related symptoms as secondary rather than primary outcomes; for example, studies evaluating pain outcomes found secondary improvements in sleep quality [26,63,64].

Evidence for the role of cannabis-based medicines for improving sleep in patients with cancer is mixed. In a randomized controlled trial primarily assessing the tolerability of high-dose THC in patients with glioblastoma, patients receiving high-dose THC also had improved sleep and significant improvements in both functional and physical domains [65]. Positive outcomes have been noted in certain studies in non-cancer population. In a post hoc analysis of a randomized double-blind study assessing the effects of nabilone on sleep outcomes in study participants, nabilone had beneficial effects on sleep outcomes in Parkinson’s disease patients that had baseline sleep disorders [66]. In another randomized, double-blind crossover trial aimed at comparing nabilone to amitriptyline among patients diagnosed with fibromyalgia and chronic insomnia, the primary objectives centered on assessing the quality of sleep using measures such as the Insomnia Severity Index and the Leeds Sleep Evaluation Questionnaire. The study revealed that nabilone effectively enhanced sleep among individuals with fibromyalgia and chronic insomnia, and demonstrated good tolerability [67]. In a randomized, double-blind, placebo-controlled, graded-dose study, individuals with advanced cancer and pain refractory to opioids were administered either placebo or Nabiximols at varying doses: low, medium, or high. Throughout the five-week treatment period, average pain, worst pain, and sleep disruption were monitored daily. Patients receiving the low and medium doses of Nabiximols reported improvements in sleep [23]. In two double-blind, randomized, placebo-controlled phase 3 trials involving patients with advanced cancer and chronic pain, where patients would be assigned to receive Sativex or placebo in one study and self-titrate Sativex in the second study, The Sleep Disruption Numerical Rating Scale was assessed as a secondary efficacy endpoint in both studies. No significant differences in sleep disruption scores were observed between the Sativex and placebo groups in either trial [25]. In a Phase 3, double-blind, randomized, placebo-controlled trial involving patients with advanced cancer experiencing average pain with Numerical Rating Scale scores between ≥4 and ≤8 despite receiving optimized opioid therapy, individuals were randomly assigned to receive either Nabixmols or a placebo. Participants self-titrated the study medications over a two-week period, followed by a three-week treatment phase at the titrated dose. The secondary endpoint of the study was the Sleep Disruption Numerical Rating Scale. Although the primary efficacy endpoint did not show a preference for Nabiximols in terms of pain scores, Nabiximols did exhibit a lower sleep disruption score compared to the placebo [26]. In a randomized double-blind clinical trial involving lung cancer patients diagnosed with anorexia, the efficacy of Nabilone versus a placebo was investigated using the Anorexia Cachexia/Anorexia Cachexia Subscale (AC/S) of the Functional Assessment of Anorexia Cachexia Therapy tool. The study revealed that patients in the Nabilone group experienced improvements in insomnia, while no significant changes were observed in the control group [55].

In a secondary analysis of data focusing on oncology patients utilizing medical cannabis, a study employing semi-structured interviews was conducted. The participants included patients with documented cancer history and legal authorization to access medical cannabis. According to patient reports, medical cannabis was associated with enhancements in both sleep onset and continuity, accompanied by a reduction in the use of sleep medications. However, one limitation of the study pertains to the recruitment method, as patients were solicited through advertisements placed at cannabis dispensaries and had been using medical cannabis [61].

Improvement in sleep was not demonstrated in other studies [23,29,68,69,70]. During a two-week trial conducted across multiple centers, employing a double-blind, randomized, placebo-controlled design with parallel groups, researchers investigated the effectiveness of THC:CBD extract and THC extract compared to a placebo in managing pain among oncology patients experiencing moderate to severe cancer-related pain and not attaining sufficient pain relief from opioids. Among the secondary endpoints examined was the evaluation of sleep quality by patients, measured using a numerical rating scale for mean sleep quality. The study found no notable variances in sleep quality when comparing the THC:CBD extract or THC extract groups with the placebo group [22]. In a randomized, double-blind, placebo-controlled trial conducted at a single center, the effectiveness of Nabilone versus a placebo group was investigated in patients undergoing radiotherapy for head and neck cancer. Nabilone or placebo administration commenced prior to radiotherapy, with dosage escalation following a fixed protocol during weeks 1 and 2. From week 3 until the completion of radiotherapy, dosage adjustments were made by the radio-oncologist. The primary end-point measure was quality of life measured using the EORTC QLQ-C30 Quality of Life Questionnaire. The study revealed no discernible variance in patients’ sleep patterns during the treatment period [69].

Similar to the approach to management of other symptoms, disorders of sleep can be a result of multiple etiologies. Proper assessment, consideration of differential diagnosis, and targeted management of the most likely etiology should be employed [71]. The choice of pharmacologic and non-pharmacologic management for sleep would be based on these assessments, including if the addition of cannabis-based medicines is warranted.

In patients with cancer, treatment of mood disorders, such as anxiety and depression, with cannabinoids is not as well-studied as the treatment of mood disorders with first-line psychotherapy and pharmacologic therapies. In a trial investigating Nabiximols for the treatment of chemotherapy-induced neuropathic pain, the Nabiximols group had greater improvements in physical and mental subscales for quality of life than the placebo group [10]. In studies by Ungerleider et al., patients receiving THC did not show significant improvement in mean scores for anxiety and depression compared with patients receiving prochlorperazine [72,73]. Interestingly, patients who previously used illicit substances reported lower anxiety while receiving THC than patients who had not previously used illicit substances [74]. In other studies, quality-of-life scores were not significantly better for patients receiving dronabinol or THC than for patients receiving a placebo [53,54].

## 6. Adverse Effects and Potential Harms

Various adverse effects associated with the use of cannabis have been reported in the literature (Table 2) [22,41,43,44,45,46,73,74,75,76,77,78,79,80,81,82,83]. Some of the more commonly reported adverse effects include dizziness, dry mouth, nausea, fatigue, somnolence, and euphoria [84]. While most adverse effects from cannabis-based medicines were reported to be mild to moderate, certain studies reported the adverse effects were significant enough to lead to study withdrawals [27,44,47,75,81]. Although adverse effects are not the focus of this paper, we wanted to highlight certain harms that can impact symptom management, especially in palliative care patients, who for the most part are significantly unwell and already taking multiple medications that result in similarly appearing adverse reactions.

### 6.1. Neuropsychiatric Effects

In heavy cannabis users, structural changes may develop in the brain, including altered patterns of brain activity, particularly around the limbic and prefrontal areas [78,79,80,85]. Studies have found impaired reaction time, cognition, and psychomotor function in patients receiving cannabis-based medicines [81]. These impairments explain why cannabis use is associated with an increased risk of motor vehicle collisions [86,87]. Furthermore, cannabis use has been associated with an increased risk of psychologic disorders, including increases in anxiety and depressive symptoms, and even triggering manic episodes in those with bipolar disorder [76,82,83].

Cannabis use disorders occur in approximately 10% of cannabis users [88]. Moreover, people with cannabis use disorders are more likely to have a concomitant substance use disorder [89,90].

### 6.2. Systemic Effects

Cannabis-based medicines have been associated with a myriad of adverse systemic effects. Inhalation of cannabis has been shown to result in inflammation and structural changes in airways, as well as increased rates of symptoms such as cough, wheezing, and sputum production [76,91,92]. A review also described increased incidences of myocardial infarction, stroke, and sudden cardiac death in cannabis users [93].

Although cannabis can be used for the treatment of nausea and vomiting, a paradoxical condition known as cannabis hyperemesis syndrome may occur. This syndrome usually occurs in chronic cannabis users and is characterized by prodromal, hyperemesis, and recovery phases [94,95,96]. The prodromal phase is typified by early morning anticipatory nausea and vague abdominal discomfort. The prodromal phase is followed by the hyperemesis phase, which is characterized by diffuse abdominal pain with severe cyclical nausea and vomiting [95,96]. The final phase is the recovery phase, in which your body begins to return to normal. Frequent hot baths are usually the most effective treatment to reduce the symptoms of hyperemesis syndrome, and cessation of cannabis is the only definitive treatment [96,97,98].

### 6.3. Drug Interactions

Both THC and CBD are metabolized via cytochrome P450 (CYP) enzymes CYP2C9, CYP2C19, and CYP3A4, while CBD can also be metabolized by CYP1A1, CYP1A2, and CYP2D6 [99]. Cannabis use may result in inhibition of CYP2C9, CYP2B6, and CYP2C19 and partial inhibition of CYP3A4, but data on the potential role of cannabis as an inhibitor of CYP2D6 are mixed [100,101,102]. These CYP enzymes are responsible for the metabolism of various medications, and inhibition of these enzymes result in decreased efficacy of the drugs or increased drug toxicity.

Opioids are a mainstay in management of cancer-related pain, and drug interactions between opioids and cannabinoids can occur. Commonly used opioids such as fentanyl, methadone, and oxycodone are also metabolized by various CYP enzymes, including CYP3A4, CYP2C19, CYP2C9, and CYP2D6. In one case report, a 13-year-old patient with cancer who was receiving stable doses of methadone suddenly developed worsening sleepiness and fatigue after receiving increased levels of CBD [103]. Cannabis has also been shown to interact with buprenorphine, an opioid that has been increasingly used for pain management and in drug detoxification centers. Cannabis use in patients receiving buprenorphine has resulted in increased levels of buprenorphine and norbuprenorphine in the blood [104].

Cannabis may also interact with other protein transporters such as p-glycoprotein, breast cancer resistance protein, and substrates for mitoxantrone, topotecan, and vincristine [100]. Certain cancer-directed therapies including chemotherapies, hormonal treatments, and immune checkpoint inhibitors may also interact with cannabinoids [100,105,106,107,108]. Patients should be carefully screened and monitored for potential drug interactions and counseled on the potential harms of using cannabis.

## 7. Conclusions and Future Directions

While this review describes various publications examining the use of cannabis and cannabinoids in the treatment of cancer and cancer-treatment related symptoms, the narrative nature limits a more comprehensive understanding of the various methodology and results of these publications. There is currently limited evidence supporting the use of cannabis-based medicines to treat various cancer-related symptoms. Even so, patients are showing a growing interest in the use of cannabis for symptom management. Clinicians should remain steadfast in inquiring if patients use cannabis products, as knowledge of patients’ cannabis use can help clinicians recognize cannabis-related toxic effects and avoid potentially harmful drug interactions. Further research exploring the potential short- and long-term risks and benefits of cannabis-based products is needed.

## Figures and Tables

**Table 1:** Administration routes for cannabis-based products.

Routes of Administration
Inhalation of aerosolized cannabinoids (“vaping”). Vaporizers can be used with dried cannabis flower or cannabis concentrates. Inhalation of smoke produced by burning of cannabis (“smoking”). Inhalation of vaporized cannabis concentrates, such as dabbing. Oral consumption of cannabis-infused products (e.g., edibles, teas). Rectal administration of cannabis suppositories. Sublingual administration of cannabis-infused sprays. Sublingual placement of liquid extracts of cannabis (“tinctures”). Often, small amounts of extract are used. Topical application of cannabis-infused compounds (e.g., creams, balms, ointments, lotions, transdermal patches) on the skin.

**Table 2:** Adverse effects associated with cannabis use.

Category	Adverse Effects
Systemic effects	
Neuropsychiatric	- Impaired coordination and motor skills - Impaired short-term memory - Altered judgment and decision-making - Anxiety or panic attacks - Psychosis - Impaired cognition and attention - Increased risk of mental health issues - Dependence and withdrawal symptoms - Cannabis use disorders
Cardiovascular	- Tachycardia - Blood pressure changes - Increased risk of myocardial infarction (in individuals with preexisting cardiovascular conditions) - Increased risk of sudden cardiac death
Respiratory	- Irritation of the respiratory tract - Chronic bronchitis (with long-term smoking) - Coughing, wheezing, and sputum production - Increased risk of respiratory infections - Structural changes in airways
Gastrointestinal	- Xerostomia - Nausea and vomiting (especially at high doses) - Changes in appetite - Abdominal pain or discomfort - Cannabis hyperemesis syndrome
Endocrine	- Altered hormone levels (reproductive hormones) - Disrupted menstrual cycle - Increased risk of diabetic ketoacidosis in type 1 diabetics - Increased risk of prediabetes
Immune	- Suppression of the immune system
Reproductive	- Reduced fertility in both men and women - Pregnancy complications
Drug interactions	- Potential CYP450 inhibition - P-glycoprotein inhibition - Potential chemotherapy interference - Potential immune checkpoint inhibitor interference - Hormonal treatment interaction

CYP450, cytochrome P450.
